# Effect of Shearing for Improving the Thermoregulatory Responses of Crossbred Sheep During Heat Stress

**DOI:** 10.3390/vetsci12040358

**Published:** 2025-04-11

**Authors:** Lina Fernanda Pulido-Rodríguez, Alfredo Manuel Franco Pereira, Fábio Luís Henrique, Ricardo De Francisco Strefezzi, Messy Hannear de Andrade Pantoja, Daniel Mota-Rojas, Cristiane Gonçalves Titto

**Affiliations:** 1 Faculdade de Zootecnia e Engenharia de Alimentos, Universidade de São Paulo, Duque de Caxias Norte 225, Pirassununga, São Paulo 13635-900, SP, Brazil; linafernanda.pulidorodriguez@unifi.it (L.F.P.-R.); fabio.luishenrique@gmail.com (F.L.H.); rstrefezzi@usp.br (R.D.F.S.); messy.pantoja@alumni.usp.br (M.H.d.A.P.); 2 Dipartimento di Scienze delle Produzioni Agroalimentari e dell’Ambiente (DISPAA), University of Florence, 50144 Firenze, Italy; 3 Mediterranean Institute for Agriculture, Environment and Development, Institute for Advanced Studies and Research, Universidade de Évora, Pólo Mitra, Ap. 94, 7006-554 Évora, Portugal; apereira@uevora.pt; 4 Neurophysiology, Behavior and Animal Welfare Assessment, DPAA, Xochimilco Campus, Universidad Autónoma Metropolitana (UAM), Mexico City 04960, Mexico; dmota@correo.xoc.uam.mx

**Keywords:** heat stress, infrared thermography, shearing, sheep, sweat glands, thermoregulatory responses

## Abstract

Shearing to improve heat dissipation is common in sheep farming. This study evaluated the influence of shearing on thermoregulatory responses of crossbred sheep from adapted breeds exposed to heat stress. Ten ewes were kept in a climate chamber exposed to heat stress (10–13 h), reaching 37 ± 1.0 °C for nine days, during two consecutive periods, without shearing, and then at 14 days after shearing, evaluating all variables. Rectal temperature, respiration rate, sweating rate, ocular surface temperature, and skin temperature were analyzed at 7, 10, 13, 17, and 20 h, and blood cortisol was analyzed at 14 h on days 1, 5, and 9. Skin and sweat gland morphologies one time, on the last day. At 13 h, there was no difference in rectal temperature (*p* > 0.05); however, at 20 h, sheared ewes had higher values (*p* ≤ 0.05). Ocular surface temperature and skin temperatures were higher after shearing (*p* ≤ 0.05). At 13 h, both control and post-shearing groups presented a higher respiration rate (*p* > 0.05). Sweating rate was higher in unshorn animals (*p* ≤ 0.05). The glandular area was similar between treatments, and the sweat glands tended to be located more superficially. In conclusion, shearing crossbred ewes did not improve thermoregulatory responses when exposed to heat stress. In unshorn sheep, the homeothermy mechanisms are more efficient, ensuring a superior level of coping in tropical environments.

## Simple Summary

Shearing is a common practice to improve heat losses in wool sheep. However, little is known about this practice in hair x wool crosses. This study tested how sheep change thermoregulation and could improve heat loss after shearing. At fourteen days after shearing, thermal regulation did not improve in sheep exposed to consecutive heat stress episodes from 10 to 13 h, reaching 37 ± 1.0 °C of air temperature. The ocular surface temperature and skin temperatures were higher after shearing, indicating that wool could be a barrier to heat acquisition, which could explain the higher sweating rate in unshorn animals caused by the difficulty of convection. The glandular area was similar between treatments, and the sweat glands had a tendency to be located more superficially. This research contributes to the management of sheep production systems, as crossbreed unshorn sheep presented similar thermoregulatory responses as sheared ones.

## 1. Introduction

Climate change alters the world’s environmental and climatic patterns, affecting agricultural production [1] and increasing global temperatures by an average of 1 °C [2]. The climate factors that mainly influence animal production are ambient temperature, relative humidity, solar radiation, and wind speed, which are likely to increase steadily, particularly in the tropics [3,4]. Heat stress is the greatest challenge faced by animals raised in these areas. Therefore, it is essential to improve production systems and breeding strategies to compensate for the energy expenditure necessary to maintain homeothermy in areas with extreme environmental conditions [4,5,6].

Sheep production in Brazil is mainly based on the crossbreeding of native animals, such as the Santa Inês breed, which is known for its tolerance to hot climates and its fast-growing offspring, particularly when crossed with Dorpers [7,8]. Dorpers, an exotic breed from South Africa, are characterized by a short hair coat on the belly and limbs, with a small amount of wool on the upper body [9]. While both Dorper and White Dorper are hair-sheep breeds, they also produce crossbred animals with wool, except for bellies and sometimes heads [10]. Usually, Santa Ines crossbred sheep are efficient at dissipating heat using the elevation of respiration and sweating rates, despite the short wool cover [11,12].

Thermal balance in sheep is primarily influenced by the physical characteristics of the skin, including the size and number of sweat glands, epidermal and dermal thickness, and vascularization, which differ according to breed and environmental factors [13]. Respiratory evaporation is the primary pathway used to establish homeothermy [14,15]. However, despite conflicting evidence on cutaneous evaporation, this mechanism has been recognized as an important factor in heat loss in hair sheep raised in hot environments [16].

Shearing management influences the thermoregulatory responses of wool breeds [17]. Wool removal reduces thermal insulation, leading to greater heat loss and decreased rectal temperature [18,19]. Additionally, shearing can result in a decrease in surface temperature [20], favoring the thermoregulation of wool breeds in tropical environments. In contrast, in crossbred sheep farming, where meat is the primary product and wool is secondary, shearing is considered an important hygiene practice. This helps to increase the feed intake and promotes heat dissipation [21].

However, in sheep that are native to warm regions, the morphological characteristics of wool as a thermal insulator help protect against direct radiation, with a close connection to the water balance, facilitating water conservation [22,23]. The wool on the back and loin of sheep can be used as a strategy to shield themselves from radiation, improve their thermal regulation by preventing heat absorption, and act as a protective barrier for the skin. This contrasts with the practice of shearing, which is intended to enhance an animal’s ability to regulate temperature [24].

Therefore, it is important to understand how sheep interact with their environment and the role of shearing as a strategy for mitigating heat stress in crossbred sheep. This study aimed to evaluate the influence of shearing on thermoregulatory responses of crossbred Santa Inês sheep exposed to heat stress. It was hypothesized that after shearing, crossbred sheep adapted to a tropical climate do not exhibit increased heat tolerance.

## 2. Materials and Methods

### 2.1. Study Site and Period and Ethics Approval

The experiment was carried out during the summer from December 2017 to February 2018 in the climate chamber of the Department of Animal Reproduction, School of Veterinary Medicine and Animal Science (VRA/FMVZ), University of São Paulo, Fernando Costa Campus, Pirassununga (21°57′12″ S and 47°27′06″ W, altitude of 605 m). According to the Köppen classification, the region has a humid subtropical climate with predominantly summer rainfall. The Research Ethics Committee of the School of Animal Science and Food Engineering, University of São Paulo (FZEA-USP), approved all procedures described (Protocol number 1889061217). The experiments’ design followed the 3Rs principles by using the minimum number of animals required to obtain the suitable statistical power.

### 2.2. Animals, Climate Chamber, and Climate Data

Ten non-pregnant, non-lactating, crossbred White Dorper x Santa Inês ewes, raised in Coast cross (*Cynodon dactylon* (L.) Pers) pastures with free access to shade and mineral supplementation, were used. The animals were selected based on their coat characteristics. The animals had clear skin, and the coat and wool covered the entire body, except for the head, hind legs, forelegs, and belly. The wool length ranged from 10 to 15 cm and from 1 to 1.5 cm post-shearing. The animals were never shortened before this study. The animals had a mean weight of 56.03 ± 7.46 kg, a similar body condition score of approximately 3 (moderately fat), and ages ranging from 2 to 4 years.

The total area of the climate chamber was 56 m^2^. The chamber had brick and slab walls, a cemented floor, outdoor temperature and humidity sensors, internal thermostats, and an exhaust fan (Mipal Industry Ltd., Cabreúva, Brazil). The wind speed was zero. Wooden railings covering 20% of the floor were installed. The temperature was controlled using hot air circulation. The animals had free access to water, corn silage, and mineral supplements.

The microclimate variables of air temperature (AT) and relative humidity (RH) were recorded every 15 min throughout the experiment using a data logger (HOBO^®^ U12-013 Data Logger) installed inside the climate chamber. The temperature-humidity index (THI) was calculated to evaluate the comfort of the animals using the following formula [15]:

THI = AT − {(0.31 − 0.31 RH) ∗ (AT − 14.4)}

where AT is the air temperature in degrees Celsius (°C) and RH is the relative humidity [(RH%)/100]. The values obtained were classified as follows: <22.2 = absence of heat stress; 22.2 to <23.3 = moderate heat stress; 23.3 to <25.6 = severe heat stress; and ≥25.6 = extreme heat stress.

### 2.3. Study Design, Heat Exposure, and Experimental Treatments

The study was conducted over nine days of heat stress, preceded by three days of thermoneutral adaptation, for a total of 12 days from the moment the animals entered the climatic chamber until they left it. This cycle was repeated fourteen days apart, after shearing the animals. The first three days of each phase consisted of adaptation to the environment, management, routine procedures, and social groups (days −2 and −1 of heat exposure) when the temperature within the chamber was like outside.

During heat stress, from day 1 to day 9, the ewes were exposed to an average temperature of 27.5 °C during the night and to an average temperature of 32.5 °C during the day. After the night period, the temperature of the chamber was increased gradually starting at 8 h up to a temperature of 37 ± 1.0 °C, maintained between 10 h and 13 h, and RH of 60 to 70% (THI = 37.9) along the day.

In the first phase, animals were exposed to heat stress without shearing (control group). At the end of the first 12-day period, the animals left the climate chamber and were taken to Coast cross (*Cynodon dactylon* (L.) Pers.) pastures, where they were sheared. Fourteen days post-shearing (to avoid acclimatization to heat stress during the first period in the climatic chamber), the animals returned to the climate chamber and were exposed to the same heat stress as previously described. The ewes were provided ad libitum access to water, corn silage, and mineral supplements (ad libitum) inside the chamber.

### 2.4. Biological Records and Sampling

Rectal temperature, ocular surface temperature, skin temperature, respiration rate, and sweating rate (RT, OST, ST, RR, and SR, respectively) were measured in all ewes on days 2, 3, 4, 6, 7, and 8 of heat treatment at 7, 10, 13, 17, and 20 h, respectively. The SR was measured at 7, 13, and 20 h. Blood samples were collected at 14 h on days 1, 5, and 9 of heat stress by varying the day to avoid the influence of stress caused by restraint of the animal on cortisol concentration. Histological skin samples were taken three times: the first 15 days prior to the beginning of the control period, and the second and third samples after the end of each experimental treatment (day 10 of each heat treatment for unshorn ewes, then after shearing).

#### 2.4.1. Ocular Surface Temperature and Skin Temperature

**Figure 1:**
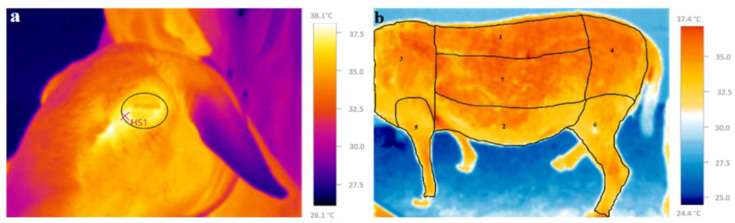
Thermographic images. (**a**) eye region, and (**b**) different body regions of sheep defined for analysis: back and loin (1), belly and flank (2), shoulder (3), rump (4), forearm, knee, and cannon (5), leg, shank, and dewclaw (6), and middle (7).

Ocular surface temperature (OST) and skin temperature (ST) were measured simultaneously with the respiration rate (RR) and immediately before rectal temperature (RT). Thermograms of each animal were obtained using a thermal imaging camera (Testo 875-2i, Baden-Württemberg, Germany). According to the manufacturer, the camera has an accuracy of ±2 °C of the real temperature, thermal sensitivity (NETD) < 50 nK, infrared resolution of 160 × 120 pixels, and image resolution of 640 × 480 pixels, with an emissivity coefficient of 0.98 at less than 1 m from the animal’s body. The images obtained were analyzed using the Testo IRsoft 3.6 software. For the measurement of OST, a circle was drawn delimiting the eye between the upper, middle, and lower borders and the lacrimal caruncle, and the maximum temperature was recorded (Figure 1a) [25,26,27]. The ST was obtained by dividing the body image into seven areas and recording the mean temperature in each area [28]. The following areas were analyzed: back and loin (1); belly and flank (2); shoulder (3); rump (4); forearm, knee, and cannon (5); leg, shank, and dewclaw (6); and middle (7) (Figure 1b).

#### 2.4.2. Rectal Temperature and Respiration Rate

Rectal temperature (RT; °C) and respiration rate were measured using a digital clinical thermometer (Geratherm^®^ rapid GT-195-1, Germany; precision ±0.1 °C) inserted 5–6 cm into the rectum of the animal in contact with the mucosa. The respiration rate (RR, breaths.min^−1^) was measured by counting the respiratory movements observed on the animal’s flanks with the aid of a stopwatch for 15 s, and the seconds were then converted into minutes.

#### 2.4.3. Sweating Rate and Sample Collection for Cortisol Analysis

Six ewes were randomly chosen to estimate the sweating rate (SR; g.m^−2^.h^−1^) [29], always using the same animals in all measurements through the day. An area of 3 cm^2^ was shaved in the mid-thoracic region, approximately 20 cm below the spine, between the 10th and 11th ribs, and three paper disks soaked in cobalt chloride were directly fixed to the skin with adhesive tape. Immediately after fixation, the time elapsed until the blue disks changed to light pink was recorded. Observations were made individually by two trained examiners to avoid variations between assessments. Each disk was scored individually, and the time of color change and mean were determined in seconds. SR was calculated by using the following equation [29]: SR = (22 × 3600)/(2.06 t) = 38,446.6/t g.m^−2^.h^−1^, where t is the mean time in seconds.

Finally, blood was collected from the same six ewes for subsequent quantification of cortisol (μg.dL^−1^). Blood samples were collected by puncturing the jugular vein into 10 mL sodium heparin tubes (BD Vacutainer) with a 25 × 8 mm needle at 14 h. Blood cortisol concentration was determined by using an electrochemiluminescence immunoassay kit (Roche Cobas Cortisol Assay, Roche Diagnostics, Rotkreuz, Switzerland) following the manufacturer’s instructions. All samples whose duplicates showed a difference greater than 10% were tested again. The validation of the kits demonstrated parallel curves between the standard concentrations and serially diluted samples.

#### 2.4.4. Skin Tissue Biopsies and Histological Analysis

Biopsies were obtained from the same ewes at the same site as the SR measurements. One skin fragment per animal was collected within the previously described shaved region using an 8 mm punch (Punch Keyes, ABC, São Paulo, Brazil). Before the biopsy, the shaved region was disinfected, and 2 mL of lidocaine hydrochloride without a vasoconstrictor was applied (Xylestesin^®^, Cristália, São Paulo, Brazil). Finally, a skin sample was obtained via a punch biopsy. The wound was then sutured, and an anti-inflammatory and analgesic combination was applied (Terra-Cortril^®^, Itapevi, Brazil). Postoperative follow-up consisted of local dressing and medication application. The sutures were removed ten days after the biopsy to ensure wound healing. The collected skin fragments were immediately stored in buffered 10% formalin solution for 24 h. After this period, the samples were stored in 70% alcohol for subsequent histological analysis. Samples were processed at the Laboratory of Pathology, Department of Veterinary Medicine, FZEA-USP.

**Figure 2:**
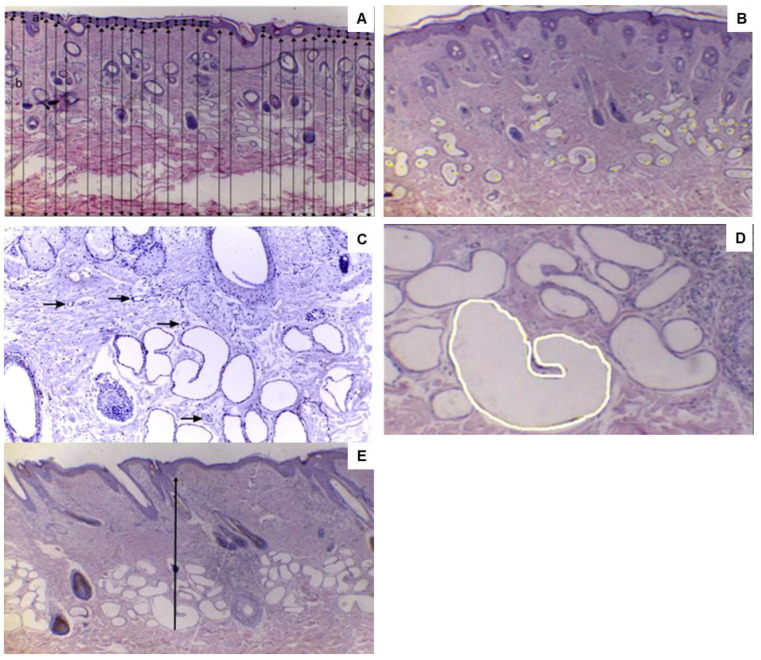
Epidermal thickness (μm) and dermal thickness (μm) (**A**), number of sweat glands (**B**), vascularization showed by arrows (**C**), glandular area (μm^2^) (**D**), and distance from the sweat gland to the epidermis (**E**) (**A**,**B**,**E**) = 4× objective; (**C**) = 10× objective; and (**D**) = 40× objective.

Approximately 4 µm-thick sections were cut from each skin fragment and stained with hematoxylin and eosin for histopathological assessment. The sections were analyzed under a microscope (Leica DM500 light, Leica Microsystems, Wetzlar, Germany) using the 4× (for epidermal and dermal thickness and distance from the sweat gland to the epidermis), 10× (for vessel counting), or 40× objectives (for glandular area). Photographs were obtained from the slides, digitizing one photograph per section (Leica ICC50-HD camera, Leica Microsystems, Wetzlar, Germany), and analyzed using the ImageJ 1.52a program (National Institutes of Health, Bethesda, MD, USA). The epidermal thickness (μm) and dermal thickness (μm) were recorded in each section, obtaining 30 measurements per slide in different parts of the epidermis and dermis (Figure 2A). The number of sweat glands (NSG) was determined by counting all glands present in an area of 3153.712 μm^2^ (0.3153 cm^2^) using a multi-point tool. The following morphological characteristics were considered for identifying sweat glands: simple tubular or saccular glands with or without a spiral duct (Figure 2B). Similarly, vascularization was evaluated by counting the blood vessels present in the areas near the sweat glands (Figure 2C). The glandular area (μm^2^) was determined using a freehand selection tool, in which the total area was obtained by drawing the area of each sweat gland (Figure 2D). The distance from the sweat gland to the epidermis was determined from the most caudal point of the sweat gland (concerning the epidermis) to the beginning of the epidermis (μm) (Figure 2E).

### 2.5. Statistical Analysis

After applying the Shapiro-Wilk test to assess normality and Levene’s test for homogeneity, the data were found to be suitable for parametric analysis, and the dependent variables were analyzed as described below.

Repeated-measures analysis of variance (ANOVA) was applied to the biological records (RT, OST, ST, RR, and SR), and each animal was considered an experimental unit. Control (before shearing) and post-shearing treatments and the different sampling times (7, 10, 13, 17, and 20 h) and their interactions were evaluated as dependent variables, and animals were also considered repeated measures and days as a repetition. Finally, the histological parameters were analyzed by repeated measures ANOVA at the three sampling times: before the animals were exposed to heat stress (baseline), after the first heat stress cycle without shearing (control), and after the second heat stress cycle post-shearing. For each characteristic, the arithmetic mean of the values was calculated for statistical analysis. For NSG, the total number of glands per sample was used. The MIXED procedure in SAS was used for all analyses, and the data were analyzed using the F test. Means were compared using the Tukey-Kramer test.

The cortisol concentration was analyzed by t-test with treatment as the dependent variable (control; post-shearing considered as the first level of repetition), and the different sampling times (day of heat treatment: 1, 5, 9) were also considered as repeated measures and their interactions.

All results are reported as mean ± standard error of the mean. The significance level was set at *p* < 0.05, while *p* values between 0.05 and 0.10 were considered as a tendency. SAS for Windows 9.4 software (Cary, NC, USA, 2016) was used for statistical analyses.

## 3. Results

### 3.1. Climatic Chamber

**Figure 3:**
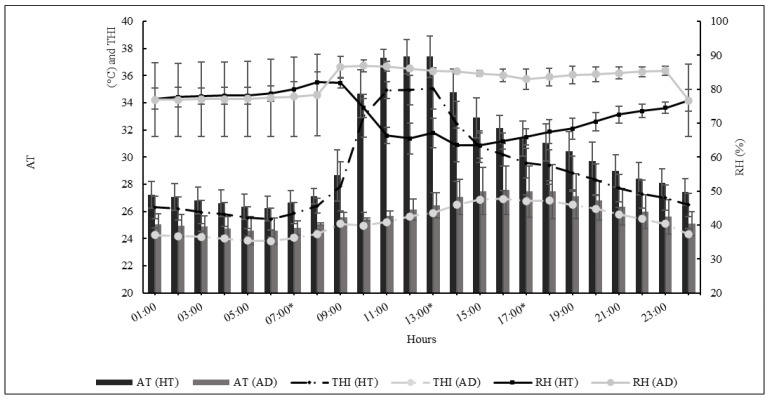
The mean and standard deviation of air temperature (AT), relative humidity (RH), and temperature-humidity index (THI) were recorded every hour during the adaptation (AD) and heat treatment (HT) periods inside the climatic chamber for both the control and post-shearing periods (*p* > 0.05) 07 h, 13 h, 17 h * biological records.

Figure 3 shows the mean AT, RH, and THI recorded every hour over the days of adaptation and during the experimental period inside the climatic chamber, indicating the intensity of heat treatment and discomfort experienced by the ewes. No interaction was found between hours during the day and the experimental period, so both control and post-shearing treatments presented similar conditions. The THI and AT peaks occurred between 11 h and 13 h, corresponding to the most significant discomfort of ewes caused by extreme heat.

Outside the climatic chamber, the animals were experiencing hot summer days, with air temperatures above 35 °C (Table 1).

### 3.2. Biological Records: Rectal Temperature, Ocular Surface Temperature, Respiration Rate, and Sweating Rate

The influence of heat treatment on biological records was evident and demonstrated by the interaction between treatment, day of heat treatment, and sampling time (*p* ≤ 0.05) (Table 2; Supplement Table S1).

During the control period, ewes increased their RT during the day by up to 0.4 °C, while this increase was 0.7 °C in the post-shearing period (*p* ≤ 0.05). However, RT did not return to baseline values in either treatment group (*p* ≤ 0.05). During the control and post-shearing heat periods, the mean RT was always similar (*p* > 0.05), but not at 20 h, when post-sheared animals presented higher RT (*p* ≤ 0.05).

During the control period, OST increased with increasing AT (from 10 h). Peak values were observed at 13 h in both treatments (*p* ≤ 0.05); however, post-shearing animals presented higher values throughout the day (*p* ≤ 0.05). The OST variation during the day was notorious, increasing by 0.7 °C in the control period and by 0.6 °C post-shearing. However, after the hottest hours of heat treatment, the animals were able to reduce the OST by up to 0.6 °C in the control period and 0.5 °C post-shearing (*p* ≤ 0.05), but the temperature did not return to baseline values during the day in either treatment.

The effect of AT on RR was significant for both treatments (*p* ≤ 0.05). During the control period, the RR of the animals increased to 43% at an AT of 32 °C (10 h), while this increase was 23% post-shearing in the same AT range (*p* ≤ 0.05). In both treatments, ewes exhibited the largest respiration rate at 13 h, corresponding to a mean increase of 124% in the control period and 168% in the post-shearing period (*p* ≤ 0.05). In the control period, the mean RR decreased by 35% and 47% four and seven hours after peak heat stress, respectively, whereas this decrease was up to 36% and 39%, respectively, over the same time interval post-shearing. Unshorn ewes exhibited the highest mean SR value (*p* ≤ 0.05). Peak SR was observed at 13 h in both the control and post-shearing periods, with a mean rate of 515 g.m^−2^.h^−1^ in the control period and of 258.2 g.m^−2^.h^−1^ post-shearing.

### 3.3. Skin Temperature

The heat treatment influenced the skin temperature in all regions (*p* ≤ 0.05; Table 3). In both treatments, ST peaks were observed in all regions at 13 h. The mean ST was up to 1.4 °C higher in all regions of sheared animals than in the control, with higher mean values in the dorsal, shoulder, and rump regions of post-sheared animals (*p* ≤ 0.05). In addition, the ST increased from 7 h to 13 h for both treatments in all regions.

Although the increase in skin temperature was similar between treatments, the decrease in ST was different. During the control period, the ST decreased significantly from 17 h to 20 h for all regions, but post-shearing values always presented similar means at 17 h and 20 h (*p* > 0.05) in all regions. Data along the days is in the Supplement File (Table S1).

### 3.4. Histological Characteristics and Hormone Response

Heat treatment did not influence the skin histological response of crossbred ewes in either treatment (Table 4). No effect on cortisol concentration (*p* > 0.05) was found between the animals in the control and post-shearing periods (Table 5). The mean cortisol concentration was higher in the control period (1.47 ± 0.22 µg.dL^−1^) than in ewes post-shearing (1.28 ± 0.19 µg.dL^−1^).

## 4. Discussion

The hypothesis that shearing could improve thermoregulation owing to changes in sweating glands and vascularization was not supported by the present findings.

THI values indicate an alert status for ewes caused by severe stress [15]. The influence of THI is evident in the animals’ physiological responses to stress before and after shearing.

It was assumed that, in sheep production systems, shearing management (besides being a hygiene-sanitary practice) could improve heat dissipation and increase feed intake [17]. An improvement in heat dissipation was expected fourteen days post-shearing; however, the physiological responses observed in this study were similar before and after shearing.

Circadian variations in the thermoregulatory responses of sheep are constant in hot environments; however, the return of physiological characteristics to normal values and the maintenance of basal levels are essential for efficient animals in terms of heat management and adaptation to high-temperature climates [15,30]. In addition, even without thermal stress, the circadian pattern of skin temperature has been well described in other studies [31,32].

The RT recorded during the two treatments was within the limits reported for the species [15,33] and was significantly higher during the hotter period. Although the RT of the animals did not return to baseline values after heat stress, an increase in RR resulted in a reduced RT. Although the results of the biological records were similar in both treatments, during the control period ewes needed to increase the RR in advance; however, the ability of these animals to maintain the RR after heat stress was more effective, with the animals being able to reduce the parameters by up to 47% in subsequent hours. However, under the same environmental conditions, respiratory movements of the animals decreased by only 39%. Respiratory evaporation is a physiological mechanism used in response to short periods of intense heat and is the first visible response in sheep to restore homeothermy [34,35].

The increase in OST coincided with the increase in AT. Both parameters simultaneously reached their maximum values. Unlike RT, the OST decreased over the hours following peak heat stress in both treatments. Although the correlation between OST and RT is high and positive in other species [26,27,36], OST follows a different pattern from that of RT in sheep. OST in sheep is influenced by the countercurrent heat exchange structure of the brain, which is attributed to a carotid network in which the blood from the artery, before entering the brain, exchanges heat by conduction with venous blood from the colder nose, providing a more suitable temperature and preventing overheating of the brain [33]. The relationship between OST and changes in the thermoregulatory system has been described by several authors [26,37], but there are no reports of OST ranges in sheep that indicate alterations in their homeothermy. Thus, studies are needed to determine the ambient temperature ranges that indicate changes in OST and the inflection point that can compromise homeothermy and animal comfort.

Shearing is expected to facilitate sensible heat loss in unshorn animals. However, shearing caused an increase in the overall skin temperature of up to 1.1 °C. The most significant increase was observed in the rump area (up to 1.4 °C). In the back + loin, leg + shank + dewclaw, middle, and rump regions, post-shearing ewes exhibited higher temperatures at 13 h, 17 h, and 20 h. In other regions, the temperature was higher at a peak time of 13 h. These animals are likely to experience heat stress and respond by increasing vasodilation to dissipate excess heat, which increases the blood flow to the skin surface [38,39]. This process increases the skin temperature, facilitates heat transfer from the body core to the skin, and supports evaporative heat loss mechanisms, all of which contribute to the regulation of core temperature [40]. This heat dissipation process may explain the higher skin temperatures observed in the post-shearing sheep.

Peripheral circulation influences the surface temperature and, consequently, sweat production [41,42]. Wool mainly acts as a thermal insulator and physical barrier that reduces short-wave radiation transmittance, preventing heat from flowing with greater intensity to the epidermis [43]. The use of thermal windows is recommended when using IRT. These regions are known as body areas with a dense number of peripheral blood vessels and, particularly, glabrous skin, such as ocular, nasal, and vulvar areas [39]. Wool, as a thermal insulator, might be a limitation for IRT to accurately record skin temperature and, therefore, to identify changes in shorn and unshorn animals.

In fact, during the control period, the ST was approximately 1.0 °C lower in regions where no wool was present, such as the ventral region, forearm, and legs, than in the other regions during hot hours. Similarly, post-shearing, the difference in temperature between these regions and the overall body was 1.3 °C, higher than that of the control. Infrared thermography permitted the evaluation of the ST by considering the heat present between the wool and skin, reinforcing the role of wool in minimizing the absorption of shortwave radiation. The present results agree with those of [44], who evaluated ST variations in Texel sheep over one year, demonstrating a lower overall ST compared with Dorper (hair sheep), Santa Inês, and Morada Nova sheep.

In contrast, the capacity of ewes to reduce their ST after 3 h of stress differed between the two treatments, with the animals losing more than 3.0 °C. However, seven hours after the AT peak, animals in the control period were more efficient in reducing the overall ST than animals in the post-shearing period. The presence of wool could act as a barrier to thermography, thus reducing the possible heat transfer [45].

The SR recorded during the control period was twice as high as that observed after shearing. In addition, unshorn ewes maintained a better ability to stabilize their RR. Although cutaneous evaporation in sheep has been widely questioned and overestimated, especially in hair sheep breeds [21,46], this mechanism is essential for the thermoregulation of animals kept and adapted to high-temperature environments [22,41]. SR, and consequently cutaneous evaporation, tend to increase with increasing fleece thickness because of the increase in epidermal temperature; thus, respiratory evaporation is less required by the animals, decreasing the respiration rate and ST [41].

Sweat glands are stimulated by the nerve endings of the glands and indirectly by the skin’s sensory fibers in response to heat stress [47,48]. Sweat is produced more frequently when animals are maintained at high temperatures [10,22,30,41,42]. Furthermore, sweat production is determined by the number of active sweat glands in animals as well as their size and depth [44,49]. Although the sweat gland characteristics did not differ statistically, the SR was lower after shearing. Despite the absence of significant differences in vascularization results, the number of blood vessels was lower after shearing. Although a smaller number of blood vessels did not influence the vasodilation response, as a more significant increase in ST post-shearing was observed, the thermolytic response by evaporation was also affected. These results might be explained not by reduced vascularization, but by the higher evaporation of sweat in the absence of wool and solar radiation, causing lower sweat measurements. In sheep with a full fleece, sweat evaporation from the skin is obstructed by air pockets filled with water vapor by wool fibers [50]. The passive movement of water vapor through the skin is minimal with a dense coat unless the hair is disturbed, as occurs during sweating rate sampling [51]. Additionally, the temporary heat generated by the absorption of sweat or moisture in the fleece can impose extra heat stress on animals [50]. Blood cortisol levels range from 1.52 to 3.2 μg.dL^−1^ in the presence of heat stress [52], while in the absence of stress, levels range from 0.6 to 1.4 μg.dL^−1^ [52,53]. Management activities in animals can cause psychological stress, influencing cortisol concentration and, consequently, affecting thermogenesis [52]; however, the low levels observed in this study suggest relatively low stress in the animals. As sheep are a very reactive species, the plasma cortisol concentrations found in the present study were maintained within baseline values for sheep, which could indicate an absence of psychological stress, which is important to guarantee optimal animal welfare in production.

Furthermore, the low cortisol concentrations recorded in this experiment demonstrated no acute heat stress, and the sheep underwent a good acclimation process. Sudden exposure to high temperatures increases blood cortisol levels in sheep; however, when the contact with the same climatic conditions is sufficiently long, a gradual decrease eventually occurs [34].

Thermoregulatory capacity may be compromised by the presence of wool, particularly in hot and humid regions [54]; therefore, shearing is a common practice to enhance heat tolerance [45]. Hair sheep and their crosses with wool breeds exhibit distinct adaptive profiles, suggesting that purebred hair sheep, having evolved in tropical environments, are better adapted to stressful production conditions than temperate wool breeds [16]. On the other hand, despite the wool cover, Santa Ines x wool-bred sheep were efficient at dissipating heat through the elevation of body surface temperature and respiratory rate [16,22].

However, shearing does not alter the functionality of thermoregulatory mechanisms [22,55]. Several studies have shown that shearing improves the physiological response of sheep when exposed to high radiant temperatures [21,51,55,56,57,58]; however, the ambient conditions need to be considered, mainly concerning radiation and relative humidity [17,59,60]. Shearing results in a marked but transient elevation in skin temperature, promoting an increase in the normal metabolism in animals [17]. However, it must be considered that the environmental conditions must be within the limits of the comfort zone for sheep. It is essential to recognize the drastic changes that are currently taking place globally and the need to estimate the acclimatization capacity of sheep before carrying out different farm practices.

One limitation of this study is that wool, as a thermal insulator, might be a limitation for IRT to accurately record skin temperature and, therefore, to identify changes in shorn and unshorn animals.

## 5. Conclusions

Shearing crossbred Dorper × Santa Inês and White Dorper × Santa Inês sheep reared in tropical regions did not improve thermoregulatory response, showing that the presence of wool is important for the protection and thermoregulation of animals, especially in the absence of direct solar radiation.

## Figures and Tables

**Table 1:** Mean ± standard error of mean of air temperature, black globe temperature, relative humidity, and temperature humidity index (THI) measured at 7 h, 13 h, and 20 h during the experiment from outside the climatic chamber.

Meteorology Data	Hour			SE	*p* Value
	7 h	13 h	20 h		
Air Temperature (°C)	21.93 ^c^	34.89 ^a^	25.54 ^b^	0.21	<0.0001
Black Globe Temperature (°C)	23.33 ^c^	43.45 ^a^	24.07 ^b^	0.45	<0.0001
Relative Humidity (%)	84.00 ^a^	61.71 ^c^	69.92 ^b^	0.44	<0.0001
THI	21.55 ^c^	32.41 ^a^	24.50 ^b^	0.15	<0.0001

Different letters at 7 h, 13 h, and 18 h represent *p* < 0.05.

**Table 2:** Mean and standard error of the mean (SEM) of rectal temperature, ocular surface temperature, respiration rate, and sweating rate measured in crossbred ewes before (control) and after shearing during heat treatment in a climate chamber.

Biological Records	Hour	Control	Post-Shearing
Rectal Temperature (°C)	7	38.43 ± 0.044 ^bA^	38.49 ± 0.052 ^cA^
	10	38.82 ± 0.041 ^aA^	38.60 ± 0.046 ^cA^
	13	38.90 ± 0.042 ^aA^	39.00 ± 0.047 ^abA^
	17	38.82 ± 0.044 ^aA^	38.92 ± 0.054 ^bA^
	20	38.89 ± 0.038 ^aB^	39.10 ± 0.051 ^aA^
Ocular Surface Temperature (°C)	7	37.76 ± 0.078 ^cB^	38.01 ± 0.059 ^cA^
	10	38.40 ± 0.062 ^bB^	38.55 ± 0.065 ^cA^
	13	39.02 ± 0.066 ^aB^	39.24 ± 0.074 ^aA^
	17	38.41 ± 0.072 ^bA^	38.57 ± 0.073 ^bA^
	20	38.49 ± 0.065 ^bB^	38.71 ± 0.085 ^bA^
Respiration Rate (movements.min^−1^)	7	49 ± 2.2 ^dA^	48 ± 2.5 ^dA^
	10	72 ± 2.9 ^bA^	59 ± 2.9 ^cB^
	13	112 ± 3.6 ^aB^	133 ± 3.7 ^aA^
	17	73 ± 3.3 ^bB^	85 ± 3.6 ^bA^
	20	59 ± 2.9 ^cB^	81 ± 3.7 ^bA^
Sweating Rate (g.m^−2^.h^−1^)	7	357.21 ± 22.175 ^bA^	195.13 ± 12.39 ^bB^
	13	806.61 ± 96.386 ^aA^	339.63 ± 53.169 ^aB^
	20	381.09 ± 25.284 ^bA^	239.91 ± 17.847 ^bB^

Means followed by different lowercase letters indicate differences in columns. Means followed by different uppercase letters indicate differences in the rows. Comparison by the Tukey-Kramer test (5% probability).

**Table 3:** Mean skin temperature (°C) measured by infrared thermography in crossbred ewes before (control period) and post-shearing during heat treatment in a climate chamber.

Body Regions	Time	Control	Post-Shearing
Back + Loin	7 h	32.1 ± 0.21 ^eA^	32.3 ± 0.19 ^dA^
	10 h	35.9 ± 0.19 ^bA^	34.3 ± 0.19 ^cB^
	13 h	37.8 ± 0.19 ^aB^	38.9 ± 0.20 ^aA^
	17 h	34.3 ± 0.19 ^cB^	34.9 ± 0.19 ^bA^
	20 h	33.1 ± 0.19 ^dB^	34.7 ± 0.19 ^bA^
Belly + Flank	7 h	34.1 ± 0.17 ^dA^	33.8 ± 0.17 ^dA^
	10 h	36.4 ± 0.18 ^bA^	35.0 ± 0.17 ^cB^
	13 h	37.6 ± 0.18 ^aB^	38.1 ± 0.17 ^aA^
	17 h	36.0 ± 0.18 ^bA^	35.9 ± 0.17 ^bA^
	20 h	35.2 ± 0.17 ^cA^	35.7 ± 0.17 ^bA^
Shoulder	7 h	33.9 ± 0.19 ^eA^	33.5 ± 0.18 ^dA^
	10 h	36.6 ± 0.18 ^bB^	35.1 ± 0.18 ^cA^
	13 h	37.9 ± 0.19 ^aB^	39.0 ± 0.18 ^aA^
	17 h	35.6 ± 0.18 ^cA^	36.0 ± 0.18 ^bA^
	20 h	35.1 ± 0.18 ^dB^	35.7 ± 0.18 ^bA^
Rump	7 h	32.2 ± 0.18 ^eB^	33.1 ± 0.18 ^dA^
	10 h	35.8 ± 0.19 ^bA^	34.6 ± 0.18 ^cB^
	13 h	37.5 ± 0.18 ^aB^	38.9 ± 0.18 ^aA^
	17 h	34.6 ± 0.19 ^cB^	35.5 ± 0.18 ^bA^
	20 h	33.8 ± 0.18 ^dB^	35.3 ± 0.18 ^bA^
Forearm + knee + cannon	7 h	33.6 ± 0.16 ^eA^	33.8 ± 0.16 ^dA^
	10 h	36.5 ± 0.17 ^bA^	34.8 ± 0.15 ^cB^
	13 h	37.6 ± 0.19 ^aA^	37.8 ± 0.16 ^aA^
	17 h	35.7 ± 0.17 ^cA^	35.9 ± 0.16 ^bA^
	20 h	34.9 ± 0.18 ^dB^	35.8 ± 0.16 ^bA^
Leg + Shank + Dewclaw	7 h	33.1 ± 0.18 ^eA^	33.3 ± 0.16 ^dA^
	10 h	36.1 ± 0.21 ^bA^	34.7 ± 0.15 ^cB^
	13 h	36.9 ± 0.23 ^aB^	37.7 ± 0.16 ^aA^
	17 h	35.2 ± 0.18 ^cB^	35.7 ± 0.15 ^bA^
	20 h	34.4 ± 0.16 ^dB^	35.7 ± 0.16 ^bA^
Middle	7 h	33.3 ± 0.18 ^eA^	33.1 ± 0.18 ^dA^
	10 h	36.3 ± 0.18 ^bA^	34.7 ± 0.18 ^cB^
	13 h	37.7 ± 0.18 ^aB^	38.9 ± 0.18 ^aA^
	17 h	35.1 ± 0.17 ^cB^	35.6 ± 0.18 ^bA^
	20 h	34.6 ± 0.18 ^dB^	35.4 ± 0.18 ^bA^

Means followed by different lowercase letters indicate differences in columns. Means followed by different uppercase letters indicate differences in rows. Comparison by the Tukey-Kramer test (5% probability).

**Table 4:** Mean ± standard error of the mean of histological characteristics of the skin and sweat glands of crossbred ewes before (control) and post-shearing during heat treatment in a climate chamber.

Characteristics	Treatment			*p* Value
	Baseline	Control	Post-Shearing	
EDT (µm)	29.96 ± 5.55	41.18 ± 5.07	36.27 ± 5.07	0.36
DT (µm)	2048.8 ± 43.63	1952.7 ± 43.89	2077.4 ± 43.89	0.08
NSG	33 ± 4.27	40 ± 4.05	35 ± 4.05	0.28
ASG (µm^2^)	16,574 ± 3102	19,265 ± 2928	15,433 ± 2928	0.37
DSG-ED (µm)	1205.8 ± 79.64	1380.9 ± 74.16	1407.8 ± 74.16	0.09
VSG	31.89 ± 2.79	30.94 ± 2.79	23.11 ± 2.79	0.08

EDT, epidermal thickness; DT, dermal thickness; NSG, number of sweat glands; ASG, area of sweat glands; DSG-ED, distance from the sweat gland to the epidermis; VSG, blood vessels present near the sweat glands.

**Table 5:** Mean ± standard error of the mean of cortisol concentration of crossbred ewes before (control) and post-shearing during heat treatment in a climate chamber.

Treatment	Day			*p* Value
	1	5	9	
Control	1.87 ± 0.46	1.49 ± 0.48	1.04 ± 0.51	0.45
Post-shearing	1.68 ± 0.46	1.24 ± 0.49	0.92 ± 0.51	0.33

## Data Availability

The data are contained within this article and Supplementary Materials.

## Supplementary Materials

The following supporting information can be downloaded at: https://www.mdpi.com/article/10.3390/vetsci12040358/s1, Table S1: Mean and standard error of the mean (SEM) of rectal temperature (RT, °C), ocular surface temperature (OST, °C), respiratory rate (RR, movements.min^−1^) and sweating rate (SR, g.m^−2^.h^−1^) measured in sheep before (control) and after shearing subjected to heat stress in a climate chamber.

## Abbreviations

The following abbreviations are used in this manuscript:

OST	Ocular surface temperature
ST	Skin temperature
RR	Respiration rate
RT	Rectal temperature
SR	Sweating rate
NSG	Number of sweating glands
AT	Air temperature
RH	Relative humidity
THI	Temperature humidity index
