# Supplementary material for: Effect of Shearing for Improving the Thermoregulatory Responses of Crossbred Sheep During Heat Stress
Source: Vet Sci. 2025 Apr 11;12(4):358. doi: 10.3390/vetsci12040358 (PMC12031516; doi:10.3390/vetsci12040358)
Supplement: Supplementary file 1 [file vetsci-12-00358-s001.zip › vetsci-3526692-supplementary.pdf]

Table S1 – Supplement: Mean and standard error of the mean (SEM) of rectal temperature (RT, °C), ocular surface temperature (OST, °C), respiratory rate (RR, movements.min^{-1}) and sweating rate (SR, g.m^{-2}.h^{-1}) measured in sheep before (control) and after shearing subjected to heat stress in a climate chamber.

		**Control**						**Post-Shearing**						**SEM**
	**Day/time**	2	3	4	6	7	8	2	3	4	6	7	8	
**RT**	07h	38.5^{Ba}	38.5^{Ba}	38.4^{Ca}	38.4^{Ba}	38.4^{Ba}	38.3^{Bb*}	38.5^{Ba}	38.4^{Bb}	38.3^{Cb}	38.3^{Bb}	38.6^{Ba}	38.7^{Ba*}	0.11
	10h	39.1^{Aa*}	38.8^{Ab*}	38.7^{Bb}	38.8^{Ab*}	38.5^{ABc}	38.8^{Ab}	38.8^{Aa*}	38.5^{Bb*}	38.5^{Cb}	38.5^{Bb}	38.6^{Bab}	38.8^{Ba}	
	13h	39.1^{Aa}	38.9^{Aab}	39.0^{Aab}	38.8^{Ab}	38.6^{ABb*}	38.8^{Ac*}	39.0^{Aa}	39.0^{Aa}	38.9^{Ba}	39.0^{Aa}	39.1^{Aa*}	39.1^{Aa*}	
	17h	38.9^{Aa}	38.5^{Bb}	38.8^{Ba}	38.8^{Aa}	38.8^{Aa}	38.9^{Aa}	39.0^{Aa}	39.0^{Aa}	38.8^{Ba}	39.0^{Aa}	39.0^{Aa}	39.0^{Aa}	
	20h	39.0^{Aa}	38.5^{Bb*}	38.7^{Bb*}	39.0^{Aa}	38.8^{Aa}	39.0^{Aa}	39.0^{Aa}	39.0^{Aa*}	39.0^{Aa*}	39.1^{Aa}	39.1^{Aa}	39.0^{Aa}	
**OST**	07h	37.8^{Ca}	38.0^{Ba*}	37.1^{Db*}	37.4^{Db*}	38.1^{Ba*}	38.0^{Ca}	38.0^{Cb}	38.3^{Ca*}	38.1^{Cab*}	38.0^{Cb*}	37.7^{Cb*}	38.0^{Db}	0.14
	10h	39.0^{Ba*}	38.1^{Bb}	38.0^{Cb}	38.3^{Cb*}	38.4^{Bab}	38.6^{Ba*}	38.3^{Ba*}	38.4^{BCa}	38.2^{Cab}	37.5^{Dc*}	38.3^{Ba}	38.0^{Db*}	
	13h	39.2^{Aa}	39.0^{Aa*}	39.0^{Aa*}	38.5^{Bb*}	39.0^{Aa*}	39.1^{Aa}	38.8^{Ac}	39.3^{Aab*}	39.2^{Ab*}	39.1^{Abc*}	39.6^{Aa*}	39.2^{Ab}	
	17h	38.6^{Bab}	37.6^{Cc*}	38.4^{Bab}	38.4^{BCb}	38.8^{Aa}	38.5^{Bab}	38.4^{Ba}	38.4^{BCa*}	38.8^{Ba}	38.6^{Ba}	38.6^{Ba}	38.4^{Ca}	
	20h	38.6^{Ba}	37.7^{Cb*}	38.1^{BCb*}	38.7^{Ba}	38.8^{Aa*}	38.7^{Ba}	38.6^{Aa}	38.7^{Ba*}	39.0^{ABa*}	38.7^{Ba}	38.3^{Bb*}	38.8^{Ba}	
**RR**	07h	53^{Ca}	57^{Ba}	37^{Cb}	48^{Cab}	52^{Ca}	50^{Cab}	44^{Dbc}	68^{Ca}	53^{Db}	37^{Dc}	45^{Cbc}	46^{Dbc}	7.05
	10h	94^{Ba*}	61^{Bb}	58^{Bb}	68^{Bb*}	72^{Bb}	85^{Bab*}	52^{Db*}	75^{Ca}	76^{Ca}	47^{Db*}	55^{Cb}	53^{CDb*}	
	13h	133^{Aa}	110^{Ab*}	112^{Ab*}	107^{Ab}	92^{Ac*}	114^{Ab}	124^{Ab}	154^{Aa*}	142^{Aa*}	122^{Ab}	134^{Ab*}	122^{Ab}	
	17h	93^{Ba}	52^{BCb*}	62^{Bb*}	63^{Bb*}	90^{Aa}	80^{Ba}	77^{Cbc}	100^{Ba*}	92^{Bab*}	85^{Bb*}	88^{Bab}	65^{Cc}	
	20h	64^{Cab*}	43^{Cc*}	41^{Cc*}	66^{Bab}	78^{ABa*}	62^{Cb*}	96^{Ba*}	98^{Ba*}	84^{Ba*}	63^{Cb}	51^{Cb*}	92^{Ba*}	
**SR**	07h	374.8^{Ba*}	371.0^{Ba}	279.8^{Ba}	281.6^{Aa}	537.5^{Aa*}	298.6^{Ba}	274.2^{Aa*}	235.4^{Aa}	218.1^{Aa}	130.1^{Aa}	145^{Ba*}	168^{Aa}	108.8
	13h	1.312.2^{Aa*}	761.9^{Ab*}	1.105.7^{Aa*}	433.7^{Ac}	523.5^{Abc}	703^{Abc*}	393.9^{Aab*}	262.2^{Ab*}	323.8^{Aab*}	276.9^{Ab}	559.3^{Aa}	221.7^{Ab*}	
	20h	323.9^{Ba}	361.6^{Ba}	355.6^{Ba}	498.0^{Aa*}	464.4^{Aa}	283.1^{Ba}	354.9^{Aa}	216.5^{Aa}	274.0^{Aa}	156.3^{Aa*}	249.1^{Ba}	188.6^{Aa}	

Means followed by different capital letters in the column differ between times within each experimental phase and day. Means followed by different lowercase letters in the row differ between days within each experimental phase and time. * differs between experimental phases within each sampling day and time. Comparison by Tukey-Kramer test at 5% probability.
